# Supplementary material for: Resolving puzzles of the phase-transformation-based mechanism of the strong deep-focus earthquake
Source: Nat Commun. 2022 Oct 22;13:6291. doi: 10.1038/s41467-022-33802-y (PMC9588062; doi:10.1038/s41467-022-33802-y)
Supplement: Supplementary file 1 — Supplementary Information [file 41467_2022_33802_MOESM1_ESM.pdf]

# Supplementary Information
# Resolving puzzles of the phase-transformation-based mechanism of the deep-focus earthquake

Valery I. Levitas

*Iowa State University, Departments of Aerospace Engineering and Mechanical Engineering, Ames, Iowa 50011, USA*

*Ames Laboratory, Division of Materials Science and Engineering, Ames, IA, USA*

## 1 Kinetics of plastic strain-induced phase transformations

The strain-controlled kinetic equation derived in [17, 18] using the main conceptual results from the nanoscale modeling of nucleation at the dislocation pileup and micromechanical treatment is

$$\frac{dc}{dq} = a\,(1-c)^s\,\frac{\sigma^w_{y2}}{\sigma_a}\,\frac{p-p^d_\varepsilon}{p^d_h-p^d_\varepsilon}H(p-p^d_\varepsilon) - b\,c^s\,\frac{\sigma^w_{y1}}{\sigma_a}\,\frac{p^r_\varepsilon-p}{p^r_\varepsilon-p^r_h}H(p^r_\varepsilon-p);\quad \sigma_a = c\sigma^w_{y1}+(1-c)\,\sigma^w_{y2}. \tag{S.1}$$

Here, $p$ is the pressure, $c$ is the volume fraction of a high-pressure phase, $\sigma_{yi}$ is the yield strength of $i$-th phase; $p^d_\varepsilon$ and $p^r_\varepsilon$ are the minimum pressure at which the direct strain-induced phase transformation (PT) may occur and maximum pressure at which the reverse strain-induced PT proceeds, respectively, $H$ is the Heaviside step function used to impose criteria for the direct ($p > p^d_\varepsilon$) and reverse ($p < p^r_\varepsilon$) strain-induced PTs; $p^d_h$ and $p^r_h$ are the pressures for the direct and reverse PTs under hydrostatic loading; symbols $a$, $b$, $s$, and $w$ are material parameters. Eq.(S.1) includes the possibility of direct and reverse PTs and the different plastic strain in each phase due to different $\sigma_{yi}$.

We do not consider strain-induced reverse spinel→olivine PT because the resultant nanograin spinel deforms dominantly by grain-boundary sliding, which does not produce stress concentrators inside the grains. The difference in yield strength of phases is neglected for compactness, which does not affect results essentially. The only existing experimental results for strain-induced PT kinetics have been obtained for $\alpha-\omega$ PT in Zr [31] and for hexagonal to wurtzitic PT in BN (our yet unpublished data), which were well described at $s = 1$. We will use $s = 1$ here as well, while different $s$ do not change the order of magnitude of the obtained in the main text kinetic estimates. Then Eq.(S.1) reduces to

$$\frac{dc}{d\varepsilon} = A(1-c) \qquad \text{for } p > p^d_\varepsilon(T); \quad A := a\frac{p-p^d_\varepsilon(T)}{p^d_h(T)-p^d_\varepsilon(T)} \quad \to \quad c = 1-\exp(-A\varepsilon), \tag{S.2}$$

which is Eq.(4) in the main text.

## 2 Analytical 3D solution for transformation induced plastic shear in a transformation-shear band

To model the transformation-deformation band in olivine, we consider an infinite space within which localized plastic deformation and PT occur (Fig. S1). TRIP occurs due to internal stresses caused by volume change during the PT combined with external stresses. We found simple analytical solutions for PT in a plastic shear band at small [47, 50] and large [49] strains in the 2D formulation. Here, we find the first 3D analytical solution. We consider the homogeneous state of the space and band before strain localization and phase transformations (i.e., including elastic strain) as the reference state. Then change in elastic strains during PT is small and can be neglected. Volumetric thermal strain is also minor compared to transformational strain and may be considered as included in it. Deformations outside the band are negligible, i.e., rigid space is considered. The stress-strain state within the band is homogeneous. For compactness and transparency, we will use small-strain formalism, while the final results will be valid for large plastic and small transformational strains, which is the case for $Mg_2SiO_4$ olivine→ $\gamma$-spinel PT (volumetric transformation strain for complete PT $\varepsilon_o = -0.096$) and for olivine→ $\beta$-spinel PT ($\varepsilon_o = -0.06$) [3, 51]. Note that since for $Fe_2SiO_4$ olivine→ $\gamma$-spinel PT $\varepsilon_o = -0.094$ [51], i.e., practically the same, these numbers can be used for $(Mg_x\, Fe_{1-x})_2SiO_4$ for any $x$. For germanium olivine→ $\gamma$-spinel PT, $\varepsilon_o = -0.077$ for $Mg_2GeO_4$ and $\varepsilon_o = -0.083$ for $Fe_2GeO_4$ [3, 51]. Thus, plots in Fig. 4(a) in the main

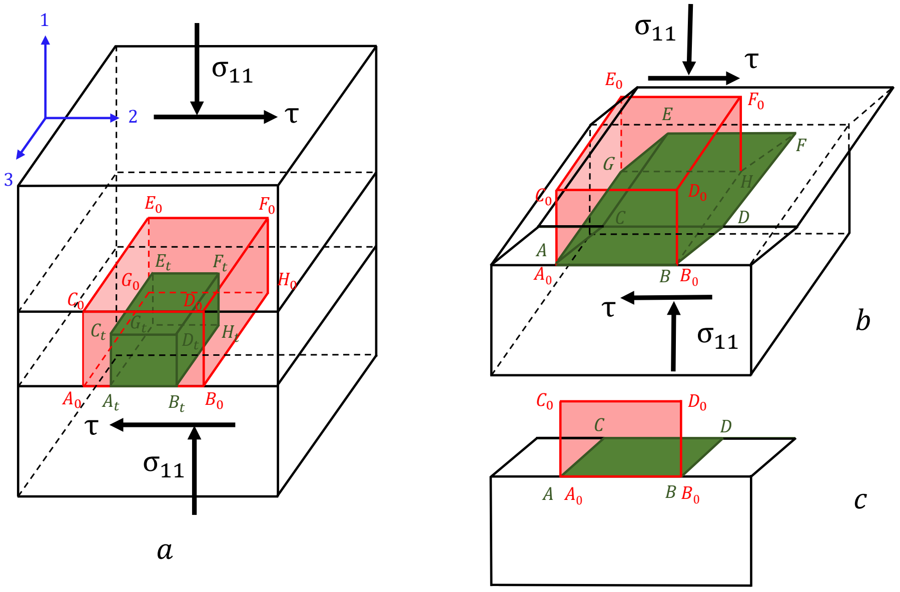


**Fig. S1: Schematics of processes in a transformation-deformation-heating band within a rigid space.** (a) Part of a band before PT (red) and after PT and isotropic transformation strain (green) is shown. (b) To satisfy the continuity of displacements across the shear-band boundary and rigid space outside the band, additional TRIP develops, leading to deformation of the green rectangular $A_tB_tG_tH_t$ to $ABGH$ that coincides with $A_0B_0G_0H_0$ and to large plastic shear. (c) 2D view (along axis 3) of (b).

text for shear strain versus $\tau/\tau_y$ between curves olivine→ $\gamma$-spinel and olivine→ $\beta$-spinel include results for all these PTs.

Let us choose the orthogonal coordinate system with axis 1 directed along the normal $\boldsymbol{n}$ to the shear band and axes 2 and 3 parallel to the shear band. We divide six components of any symmetric tensor, e.g., stress $\boldsymbol{\sigma}$, into two parts:

$$\boldsymbol{\sigma}_n := \begin{pmatrix} \sigma_{11} & \sigma_{12} & \sigma_{13} \\ \sigma_{21} & 0 & 0 \\ \sigma_{31} & 0 & 0 \end{pmatrix} = \begin{pmatrix} \sigma_{11} & \tau & 0 \\ \tau & 0 & 0 \\ 0 & 0 & 0 \end{pmatrix}; \qquad \boldsymbol{\sigma}_{||} := \begin{pmatrix} 0 & 0 & 0 \\ 0 & \sigma_{22} & \sigma_{23} \\ 0 & \sigma_{32} & \sigma_{33} \end{pmatrix}. \tag{S.3}$$

Components $\boldsymbol{\sigma}_n$ are stresses acting at the surface of the shear band, i.e., components of the traction vector; components $\boldsymbol{\sigma}_{||}$ are in-band stresses. Also, we chose axis 2 along the applied shear stress $\sigma_{12} = \tau$, i.e., $\sigma_{13} = 0$. Components $\boldsymbol{\sigma}_n$ within the band are equal to corresponding components in the space due to traction continuity conditions. Then they are equal to applied stresses and are considered independent of time during phase transformation.

The total strain $\boldsymbol{\varepsilon}^{tot}$ consists of plastic $\boldsymbol{\varepsilon}$ and transformational $\boldsymbol{\varepsilon}^t$ parts:

$$\boldsymbol{\varepsilon}^{tot} := \begin{pmatrix} \varepsilon^{tot}_{11} & \varepsilon^{tot}_{12} & \varepsilon^{tot}_{13} \\ \varepsilon^{tot}_{21} & \varepsilon^{tot}_{22} & \varepsilon^{tot}_{23} \\ \varepsilon^{tot}_{31} & \varepsilon^{tot}_{32} & \varepsilon^{tot}_{33} \end{pmatrix} = \begin{pmatrix} \varepsilon_{11} & \varepsilon_{12} & \varepsilon_{13} \\ \varepsilon_{21} & \varepsilon_{22} & \varepsilon_{23} \\ \varepsilon_{31} & \varepsilon_{32} & \varepsilon_{33} \end{pmatrix} + \begin{pmatrix} \varepsilon_t & 0 & 0 \\ 0 & \varepsilon_t & 0 \\ 0 & 0 & \varepsilon_t \end{pmatrix}, \tag{S.4}$$

where transformation strain is a spherical (pure volumetric) tensor. Decomposing Eq.(S.4) in normal and in-band parts, we obtain

$$\boldsymbol{\varepsilon}^{tot}_n := \begin{pmatrix} \varepsilon^{tot}_{11} & \varepsilon^{tot}_{12} & \varepsilon^{tot}_{13} \\ \varepsilon^{tot}_{21} & 0 & 0 \\ \varepsilon^{tot}_{31} & 0 & 0 \end{pmatrix} = \begin{pmatrix} \varepsilon_{11} & \varepsilon_{12} & \varepsilon_{13} \\ \varepsilon_{21} & 0 & 0 \\ \varepsilon_{31} & 0 & 0 \end{pmatrix} + \begin{pmatrix} \varepsilon_t & 0 & 0 \\ 0 & 0 & 0 \\ 0 & 0 & 0 \end{pmatrix}; \tag{S.5}$$

$$\boldsymbol{\varepsilon}_{||}^{tot} := \begin{pmatrix} 0 & 0 & 0 \\ 0 & \varepsilon_{22}^{tot} & \varepsilon_{23}^{tot} \\ 0 & \varepsilon_{32}^{tot} & \varepsilon_{33}^{tot} \end{pmatrix} = \begin{pmatrix} 0 & 0 & 0 \\ 0 & \varepsilon_{22} & \varepsilon_{23} \\ 0 & \varepsilon_{32} & \varepsilon_{33} \end{pmatrix} + \begin{pmatrix} 0 & 0 & 0 \\ 0 & \varepsilon_t & 0 \\ 0 & 0 & \varepsilon_t \end{pmatrix}. \tag{S.6}$$

Due to continuity of displacements across the shear-band boundary and rigid space outside the band (i.e., the coherent boundary between shear-band and the rest of the space), we obtain

$$\boldsymbol{\varepsilon}_{||}^{tot} := \mathbf{0} \quad \rightarrow \quad \boldsymbol{\varepsilon}_{||} := \begin{pmatrix} 0 & 0 & 0 \\ 0 & -\varepsilon_t & 0 \\ 0 & 0 & -\varepsilon_t \end{pmatrix}. \tag{S.7}$$

Eq.(S.7), which was derived in [48, 67], directly follows from the Hadamard compatibility condition [68] across a coherent boundary. Geometrically, it means that the boundary is undeformed and, due to homogeneity of the strain state within a band, in-band strains are absent.

It follows from Eq.(S.7) and the plastic incompressibility

$$\varepsilon_{11} + \varepsilon_{22} + \varepsilon_{33} = 0 \quad \rightarrow \quad \varepsilon_{11} = 2\varepsilon_t. \tag{S.8}$$

The von Mises yield condition

$$|\boldsymbol{S}| := \sqrt{S_{11}^2 + S_{22}^2 + S_{33}^2 + 2\sigma_{12}^2 + 2\sigma_{13}^2 + 2\sigma_{23}^2} = \sqrt{\frac{2}{3}}\sigma_y = \sqrt{2}\tau_y, \tag{S.9}$$

where $\sigma_y = \sqrt{3}\tau_y$ and $\tau_y$ are the yield strengths in compression and shear, respectively, $\boldsymbol{S} = \boldsymbol{\sigma} - \frac{1}{3}p\boldsymbol{I}$ is the deviatoric stress, and $\boldsymbol{I}$ is the unit tensor. The yield strength during PT is unknown, and based on the discussion in the main text, we will consider it a constant. Associated with the von Mises yield condition plastic flow rule is the proportionality between plastic strain rate and deviatoric stress tensors:

$$\begin{pmatrix} 2\dot{\varepsilon}_t & \dot{\varepsilon}_{12} & \dot{\varepsilon}_{13} \\ \dot{\varepsilon}_{21} & -\dot{\varepsilon}_t & 0 \\ \dot{\varepsilon}_{31} & 0 & -\dot{\varepsilon}_t \end{pmatrix} = h \begin{pmatrix} S_{11} & \tau & 0 \\ \tau & S_{22} & \sigma_{23} \\ 0 & \sigma_{32} & S_{33} \end{pmatrix}, \tag{S.10}$$

where $h$ is the proportionality factor. It follows from Eq.(S.10)

$$\dot{\varepsilon}_{13} = \dot{\varepsilon}_{31} = 0; \qquad \sigma_{23} = \sigma_{32} = 0; \qquad S_{22} = S_{33} = -0.5S_{11}. \tag{S.11}$$

Designating $\gamma = 2\varepsilon_{12}$ and utilizing Eq.(S.11), plastic flow rule simplifies to

$$\begin{pmatrix} 2\dot{\varepsilon}_t & 0.5\dot{\gamma} & 0 \\ 0.5\dot{\gamma} & -\dot{\varepsilon}_t & 0 \\ 0 & 0 & -\dot{\varepsilon}_t \end{pmatrix} = h \begin{pmatrix} S_{11} & \tau & 0 \\ \tau & -0.5S_{11} & 0 \\ 0 & 0 & -0.5S_{11} \end{pmatrix}. \tag{S.12}$$

Eq.(S.12) contains just two independent equations

$$0.5\dot{\gamma} = h\tau; \qquad 2\dot{\varepsilon}_t = hS_{11} \quad \rightarrow \quad \frac{\dot{\gamma}}{\dot{\varepsilon}_t} = 4\frac{\tau}{S_{11}}. \tag{S.13}$$

To exclude $S_{11}$, we utilize the plasticity condition Eq.(S.9)

$$\sqrt{3/2\, S_{11}^2 + 2\tau^2} = \sqrt{2}\tau_y \quad \rightarrow \quad S_{11} = sign(\dot{\varepsilon}_t)\frac{2}{\sqrt{3}}\sqrt{\tau_y^2 - \tau^2}. \tag{S.14}$$

Substituting Eq.(S.14) in Eq.(S.13), we obtain

$$\dot{\gamma} = 2\sqrt{3}|\dot{\varepsilon}_t|\frac{\tau/\tau_y}{\sqrt{1 - (\tau/\tau_y)^2}}. \tag{S.15}$$

Since during PT the volumetric transformation strain is $\varepsilon_o c$, where $c$ is the volume fraction of spinel, then $\dot{\varepsilon}_t = \frac{1}{3}\varepsilon_o \dot{c}$, and Eq.(S.15) takes its final form

$$\dot{\gamma} = \frac{2\sqrt{3}}{3}|\varepsilon_o||\dot{c}|\frac{\tau/\tau_y}{\sqrt{1-(\tau/\tau_y)^2}} \quad \rightarrow \quad \frac{d\gamma}{dc} = sign(dc)\frac{2\sqrt{3}}{3}|\varepsilon_o|\frac{\tau/\tau_y}{\sqrt{1-(\tau/\tau_y)^2}}. \tag{S.16}$$

Eq.(S.16) represents an explicit expression for plastic shear strain rate induced by PT, i.e., TRIP shear. During both direct and reverse PT, TRIP shear increases independent of the sign of the volumetric transformation strain is $\varepsilon_o$. While Eq.(S.16) has the same form as previous 2D solutions, the proportionality factor is $2\sqrt{3} \simeq 3.4$ times larger than for 2D treatment. Eq.(S.16) is used as Eq. (5) in the main text.

For completeness, pressure $p$ is determined from the equation

$$p = 3(\sigma_{11} - S_{11}). \tag{S.17}$$

## 3 Conditions for unstable heating in the shear band due to thermoplastic flow alone

Let us rewrite Eq. (3) from the main text for the stationary temperature during plastic flow alone (i.e., without PT) in the more compact form

$$T_s - T_0 = B\exp[-Q_r(T_s^{-1} - T_0^{-1})]; \qquad B = 0.25h^2\sigma\dot{\varepsilon}(T_0)/k. \tag{S.18}$$

As it follows from Fig. 3(b) in the main text and Fig. S2 here, for small $B$ Eq.(S.18) has two solutions: one of the solutions with $T \simeq T_0$ is stable and another one with $T_s \gg T_0$ is unstable. This means heating in the shear band for small $B$ is impossible without extra heat sources leading $T > T_s$. With increasing $B$, the first stable solution slightly exceeds $T_0$ while $T_s$ reduces much faster. At some critical $B = B_c$ and $T = T_c$ both solutions coincide, plastic dissipation exceeds the heat flux from the band for all temperatures (excluding $T = T_c$), the stationary solution is unstable, and unlimited heating occurs for any infinitesimal perturbation. For critical $B$, derivatives of both sides of Eq.(S.18) coincide, i.e.,

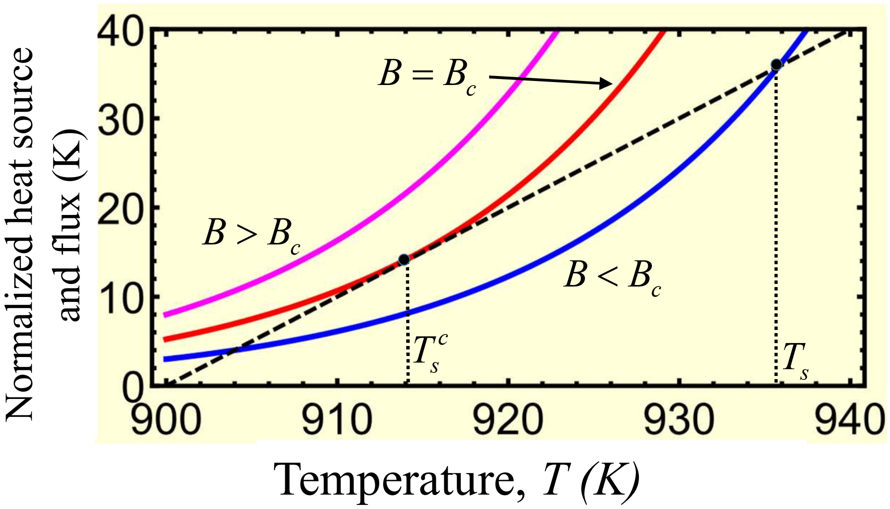


**Fig. S2: Geometric interpretation of the condition for the unstable heating in the shear band due to thermoplastic flow alone.** For relatively small parameter $B < B_c$, there are two stationary temperatures, the stable one close to $T_0$ and the unstable one $T_s$. During thermoplastic heating without PT, solution stacks in the stable stationary temperature. For the critical $B = B_c$, both stationary solutions coincide and are unstable, i.e., unlimited heating should occur. For $B > B_c$, a static solution does not exist, and unlimited heating should occur.

$$T_s^2 = BQ_r\exp[-Q(T_s^{-1} - T_0^{-1})]. \tag{S.19}$$

Excluding exponent from Eqs. (S.18) and (S.19), we obtain a simple equation

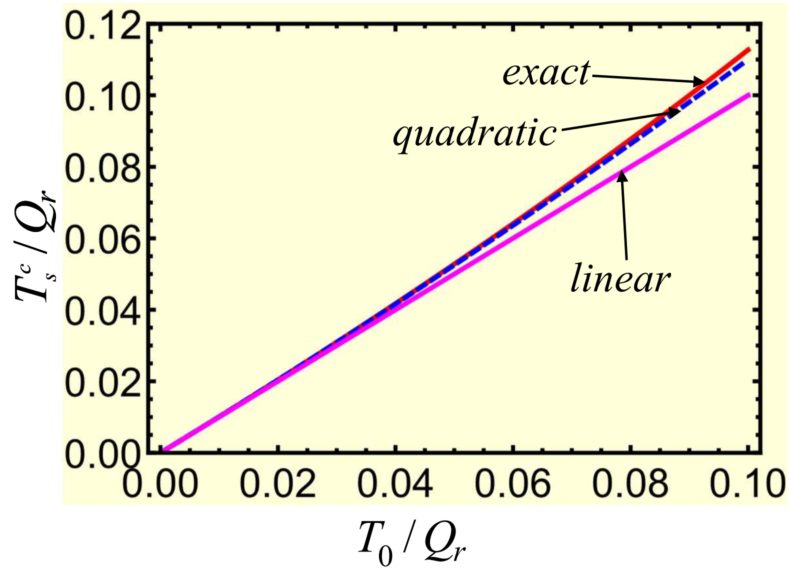


**Fig. S3: Plots for $T_s^c/Q_r$ vs. $T_0/Q_r$.** Exact dependence, as well as linear and quadratic approximations, are shown.

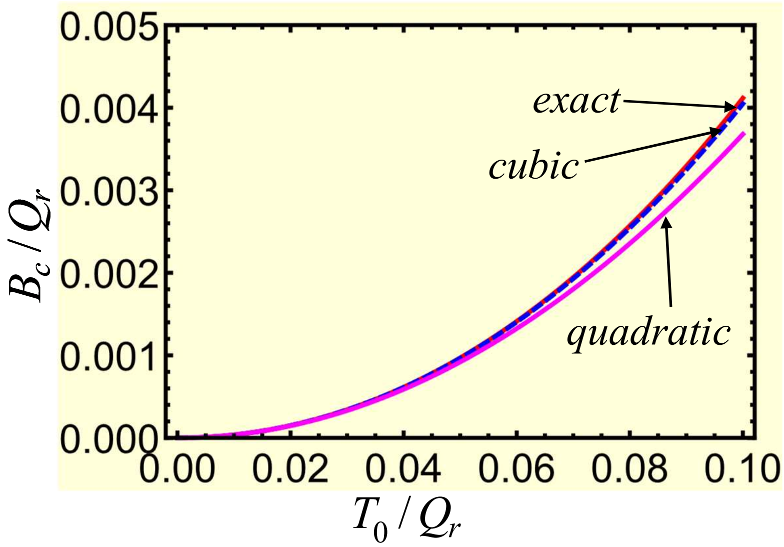


**Fig. S4: Plot for $B_c/Q_r$ vs. $T_0/Q_r$.** The exact relationship and quadratic and cubic approximations are presented.

$$T_s^2 = Q_r(T_s - T_0), \tag{S.20}$$

with the relevant solution

$$T_s^c = \frac{Q_r}{2}\left(1 - \sqrt{1 - 4\frac{T_0}{Q_r}}\right) \simeq Q_r\left[\frac{T_0}{Q_r} + \left(\frac{T_0}{Q_r}\right)^2 + 2\left(\frac{T_0}{Q_r}\right)^3\right], \tag{S.21}$$

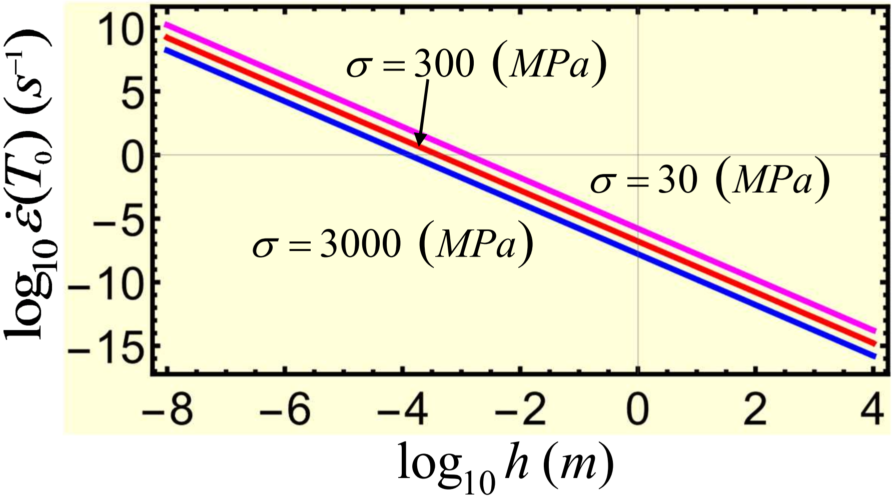


**Fig. S5: Relationships between $\log_{10}\dot{\varepsilon}(T_0)$ vs. $\log_{10} h$ for three different stresses $\sigma$.**

where due to the smallness of $T_0/Q_r$ the Taylor series is used. Substituting exact $T_s$ from Eq.(S.21) in Eq. (S.18), we obtain critical value of $B$:

$$B_c = \frac{Q_r}{2}\left(1 - \sqrt{1 - 4\frac{T_0}{Q_r}} - 2\frac{T_0}{Q_r}\right)\exp\left[-\frac{2}{1 + \sqrt{1 - 4\frac{T_0}{Q_r}}}\right] \simeq \frac{Q_r}{e}\left[\left(\frac{T_0}{Q_r}\right)^2 + \left(\frac{T_0}{Q_r}\right)^3\right], \qquad \text{(S.22)}$$

where $e = 2.718...$ is the Euler's number. Figs. S3 and S4 show plots for $T_s/Q_r$ and $B_c/Q_r$ vs. $T_0/Q_r$. It is clear that for $0 \leq T_0/Q_r \leq 0.1$, $T_s/Q_r$ is well approximated by a quadratic function and reasonably good by the linear one. The cubic approximation is not distinguishable from the exact equation. Similar $B_c/Q_r$ is very good approximated by a cubic polynomial and reasonably good by the quadratic one.

For data that we used for the Punchbowl Fault [4,29], $k = 2.4 \times 10^{-6} MPa\, m^2/(s\, K)$, $Q_r = 58,333K$, $T_0 = 900\, K$, $h = 4 \times 10^{-3} m$, $\sigma = 300\, MPa$, $\dot{\varepsilon}(T_0) = 10^{-14}$ - $10^{-10}\, s^{-1}$, we have $T_0/Q_r = 0.0154$ and $B = 5 \times 10^{-12} - 5 \times 10^{-8} K$, while $T_s = 914.33\, K$ and $B_c = 5.19\, K$, i.e., far away from the initiation of the thermoplastic instability, as expected from Fig. 3(b) in the main text.

It is convenient to present instability condition $B = B_c$ in the form

$$\log_{10}(0.25/k) + 2\log_{10} h + \log_{10}\sigma + \log_{10}\dot{\varepsilon}(T_0) = \log_{10} B_c, \qquad \text{(S.23)}$$

see Fig. S5. For the above parameters and $\dot{\varepsilon}(T_0) = 10^{-14}$ and $10^{-12}\, s^{-1}$, the instability conditions can be satisfied for $h = 4075\, m$ and $h = 407.5\, m$, respectively. These parameters are in the range obtained in [39] numerically using linear perturbation analysis. Here, simple analytical expressions are derived. For the laboratory experiment on for $Mg_2GeO_4$ from [29], $\dot{\varepsilon}_0 = 2 \times 10^{-4}\, s^{-1}$ and $\sigma = 1589\, MPa$, and the instability condition can be met for $h = 12.52\, mm$, which is still very large for the laboratory experiment.

Does this mean that shear-induced melting is impossible in Earth and the laboratory due to very small observed band thickness? Actually not. Let us assume that the initial shear band thickness can be much larger. This does not contradict to much smaller observed thickness after phase transformation (including melting) because PT leads to further softening and, due to heterogeneities, may occur in a very narrow part of the initial band. That is why after solid-solid PT or chemical reaction thickness of a band strongly decreases, and instability temperature cannot be reached at the laboratory scale (but can be met in nature). If PT or reaction does not occur below the melting temperature at a high strain rate, melting can be reached. Thus, taking $\dot{\varepsilon}_0 = 10\, s^{-1}$, $\sigma = 1000\, MPa$, we obtain critical $h = 70\, \mu m$, which may be achievable in large-volume high pressure apparatuses. Taking $\sigma = 300\, MPa$ and $h = 0.07\, m$, which may be reasonable for a shear band in nature, we obtain critical $\dot{\varepsilon}_0 = 3.33 \times 10\, s^{-5}$, which is not clear how to reach starting with $10^{-14} - 10^{-12}\, s^{-1}$ after all softening mechanisms unrelated to a PT.

## 4 Possibility of the reverse phase transformation

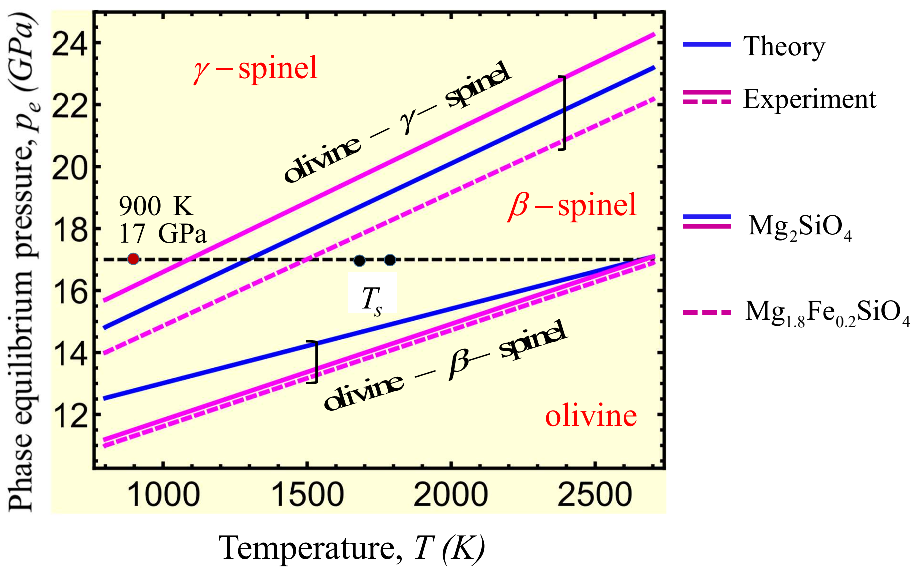


**Fig. S6: Phase equilibrium pressure-temperature diagram for olivine, $\beta$-spinel, and $\gamma$-spinel based on experiment [52] and theory [69].** Red dot designates experimental parameters for the metastable olivine, black dots correspond to the unstable stationary temperatures $T_s = 1695K$ for $\dot{\varepsilon}(T_0) = 10^{-13}\,s^{-1}$ and $T_s = 1825K$ for $\dot{\varepsilon}(T_0) = 10^{-14}\,s^{-1}$.

Here, we discuss the possibility of reaching the unstable stationary temperature $T_s$ during strain-induced olivine→spinel PT without initiating the reverse $\gamma$-spinel→$\beta$-spinel PT or spinel→olivine PT. We do not consider strain-induced reverse PT from the spinel because the resultant nanograin spinel deforms dominantly by grain-boundary sliding, which does not produce stress concentrators inside the grains. Thus, we need to analyze the possibility of the reverse pressure/temperature-induced spinel→olivine PT on the seconds time scale while direct strain-induced olivine→spinel PT occurs. The phase equilibrium diagrams from experiment [52] and theory [69] are shown in Fig. S6 for $Mg_2SiO_4$ and San Carlos olivine $Mg_{1.8}Fe_{0.2}SiO_4$. The unstable stationary temperatures $T_s = 1695K$ for $\dot{\varepsilon}(T_0) = 10^{-13}\,s^{-1}$ and $T_s = 1825K$ for $\dot{\varepsilon}(T_0) = 10^{-14}\,s^{-1}$ (Fig. 3 in the main text) are shown as well, and they are located in the region of stability of $\beta$-spinel. Thus, reverse PT to the olivine is impossible, and only reverse $\gamma$-spinel→$\beta$-spinel PT should be considered. While even without plastic straining this PT is doubtful within seconds, there is a more important argument. Strain-induced PT may occur even much below the phase equilibrium pressure [16, 18, 25, 26, 31, 65]. Since $T_s$ points are just few GPa below the olivine-$\gamma$-spinel equilibrium line, it is very probable that even if severe plastic shear of olivine would start at $T_s$, it may cause direct strain-induced olivine→$\gamma$-spinel PT. Thus, the reverse $\gamma$-spinel→$\beta$-spinel PT is not expected. Still, if it occurs, then the resulting olivine-$\beta$-spinel transformation produces slightly smaller $\varepsilon_0$, which does not change conclusions (Fig. 4a in the main text). Moreover, if $\gamma$-spinel that appeared below $T_s$ transforms back to the $\beta$-spinel ($\varepsilon_0 = 0.036$), the TRIP from both PTs will be combined, since it is determined by $|\varepsilon_0| = 0.096 + 0.036 = 0.132$.

Let us estimate the effect of nonhydrostatic stresses on the phase diagram for olivine-$\beta$-spinel. The eigen strain for this transformation, which includes transformation strain and jump in elastic and thermal strains, presented in [70] for $Mg_2SiO_4$, can be decomposed into spherical $\frac{1}{3}\varepsilon_v\mathbf{I}$ and deviatoric $\boldsymbol{e}$ parts:

$$\boldsymbol{\varepsilon}^{tr} := \frac{1}{3}\varepsilon_v \boldsymbol{I} + \boldsymbol{e} = 10^{-2}\begin{pmatrix} -2.788 & -1.021 & 0 \\ -1.021 & 3.563 & 0 \\ 0 & 0 & -7.780 \end{pmatrix} =$$

$$\frac{10^{-2}}{3}\begin{pmatrix} -7.005 & 0 & 0 \\ 0 & -7.005 & 0 \\ 0 & 0 & -7.005 \end{pmatrix} + 10^{-2}\begin{pmatrix} -0.453 & -1.021 & 0 \\ -1.021 & 5.898 & 0 \\ 0 & 0 & -5.445 \end{pmatrix}, \tag{S.24}$$

where the volumetric strain is $\varepsilon_v = -0.07005 \simeq -0.07$. The mechanical contribution to the thermodynamic driving force for phase transformation is the work of stress tensor on the eigen strain, $\sigma_{ij}\varepsilon_{ij}^{tr}$, where summation over both subscripts is assumed. This work can be decomposed into work of pressure and deviatoric stress:

$$\sigma_{ij}\varepsilon_{ij}^{tr} = -p\varepsilon_v + S_{ij}e_{ij}. \tag{S.25}$$

The upper bound of the work of deviatoric stresses, $W = |\boldsymbol{S}||\boldsymbol{e}| = \sqrt{\frac{2}{3}}\sigma|\boldsymbol{e}|$, is reached when all components $S_{ij}$ are proportional to $e_{ij}$, where the norm of the tensor is defined like in Eq. (S.9). For components $e_{ij}$ in Eq. (S.24), we obtain $|\boldsymbol{e}| = 0.0816851$ and $W = 0.0666956\sigma \simeq 0.07\sigma$. Thus

$$\sigma_{ij}\varepsilon_{ij}^{tr} \simeq 0.07(p + \sigma), \tag{S.26}$$

and nonhydrostatic stresses can lower the phase equilibrium pressure in Fig. S6 by the value of $\sigma$. Since we use $\sigma = 0.3\,GPa$ [39, 40] and at $900K$ we have $p_e = 12.77\,GPa$, the effect of nonhydrostatic stresses does not exceed 2.3%. Due small numbers and insignificant dependence of the transformation strain for $(Mg_x\ Fe_{1-x})_2SiO_4$ on $x$, the effect of the nonhydrostatic stress is small for any $x$.

Similar estimates for olivine-$\gamma$-spinel give $|\boldsymbol{e}| \simeq 0.048$, and since $\varepsilon_v = \simeq -0.096$, then

$$\sigma_{ij}\varepsilon_{ij}^{tr} \simeq 0.096(p + 0.41\sigma). \tag{S.27}$$

Since at $900K$ we have $p_e = 15.26\,GPa$, the reduction in the phase equilibrium pressure, $0.123\,GPa$, does not exceed 0.8%.

## Supplementary References


[1] Frohlich, C. The nature of deep-focus earthquakes. *Annu. Rev. Earth Planet. Sci.*, **17**, 227–254 (1989).

[2] Green, H.W. & and Burnley, P.C. A new, self-organizing, mechanism for deep-focus earthquakes. *Nature,* **341**, 773-737 (1989).

[3] Green II H. W. Shearing instabilities accompanying high-pressure phase transformations and the mechanics of deep earthquakes. *Proc. Natl Acad. Sci. USA,* **104**, 9133-9138 (2007).

[4] Green II, H. W. , Shi F., Bozhilov, K., Xia G., & Reches, Z. Phase transformation and nanometric flow cause extreme weakening during fault slip. *Nature Geoscience,* **8**, 484-489 (2015).

[5] Schubnel, A., Brunet, F., Hilairet, N., Gasc, J., Wang, Y. & Green, H.W. Deep focus earthquake analogs recorded at high pressure and temperature in the laboratory. *Science,* **341**, 1377–1380 (2013).

[6] Green, II H. W. Phase-transformation-induced lubrication of earthquake sliding. *Phil. Trans. R. Soc. A*, **375** 20160008 (2017).

[7] Kirby, S. Localized polymorphic phase transformations in high-pressure faults and applications to the physical mechanism of deep earthquakes. *J. Geophys. Res.,* **92**, 13, 789-800 (1987).

[8] Kirby, S.H., Stein, S., Okal, E. A. & Rubie, D. C Metastable mantle phase transformations and deep earthquakes in subducting oceanic lithosphere. *Rev. Geophys.,* **34**, 261–306 (1996).

[9] Zhan, Z. Mechanics and implications of deep earthquakes. *Annu. Rev. Earth Planet. Sci.,* **48**, 147-174 (2020).

[10] Wang, Y., Zhu, L., Shi, F., Schubnel, A., Hilairet, N., Yu, T., Rivers, M., Gasc, J., Addad, A., Deldicque, D., Li, Z. & Brunet, F. A laboratory nanoseismological study on deep-focus earthquake micromechanics. *Sci. Adv.* **3**, e1601896 (2017).

[11] Kawakatsu, H. & Yoshioka, S. Metastable olivine wedge and deep dry cold slab beneath southwest Japan. *Earth and Planetary Science Letters*, **303**, 1-10 (2011).

[12] Meade, C. R. & Jeanloz, R., Acoustic emissions and shear instabilities during phase transformations in Si and Ge at ultrahigh pressures. *Nature,* **339**, 616-618 (1989).

[13] Meade, C. & Jeanloz, R. Deep-focus earthquakes and recycling of water into Earth's mantle. *Science 252*, 68-72 (1991).

[14] Smart T., Li H., Dong B., Shu X., Hai R., Sun C., Zhang H. F., and Jeanloz R. High-pressure nano-seismology: Use of micro-ring resonators for characterizing acoustic emissions. *Appl. Phys. Lett.*, **115**, 081904 (2019).

[15] Officer, T. and Secco, R.A. Detection of high P,T transformational faulting in $Fe_2SiO_4$ via in-situ acoustic emission: Relevance to deep-focus earthquakes. *Physics of the Earth and Planetary Interiors*, **300**, 106429 (2020).

[16] Levitas, V.I. High-Pressure Phase Transformations under Severe Plastic Deformation by Torsion in Rotational Anvils. *Material Transactions,* **60**, 1294-1301 (2019).

[17] Levitas, V.I. Continuum Mechanical Fundamentals of Mechanochemistry. In: *High Pressure Surface Science and Engineering*, eds. Y. Gogotsi and V. Domnich, *Institute of Physics, Bristol, Section 3*, 159-292 (2004)

[18] Levitas, V. I. High-Pressure Mechanochemistry: Conceptual Multiscale Theory and Interpretation of Experiments. *Phys. Rev. B,* **70**, 184118, 1-24 (2004)

[19] Levitas, V. I. High pressure phase transformations revisited. Invited Viewpoint article. *Journal of Physics: Condensed Matter*, **30**, 163001 (2018).

[20] Burnley, P.C. & Green II, H.W. Stress dependence of the mechanism of the olivine-spinel transformation. *Nature,* **338**, 753-756 (1989).

[21] Wang, J., Yip, S., Phillpot, S.R., & Wolf D. Crystal instabilities at finite strain. *Phys. Rev. Lett.,* **71**, 4182-4185 (1993).

[22] Mizushima, K., Yip, S., & Kaxiras, E. Ideal crystal stability and pressure-induced phase transition in silicon. *Phys. Rev. B.* **50**, 14952-14959 (1994).

[23] Levitas, V.I. & Ravelo, R. Virtual Melting as a New Mechanism of Stress Relaxation Under High Strain Rate Loading. *Proceedings of the National Academy of Sciences of the United States of America*, **109**, 13204-13207 (2012).

[24] Chen, H., Levitas, V.I., Xiong, L. Amorphization Induced by $60^o$ Shuffle Dislocation Pileup against Tilt Grain Boundaries in Silicon Bicrystal under Shear. *Acta Materialia*, **179**, 287-295 (2019).

[25] Blank, V. D. & Estrin, E. I. Phase Transitions in Solids under High Pressure, *CRC Press, Boca Raton* (2014)

[26] Gao, Y., Ma, Y., An, Q., Levitas, V. I., Zhang, Y., Feng, B., Chaudhuri, J. & Goddard, III W. A. Shear driven formation of nano-diamonds at sub-gigapascals and 300 K. *Carbon,* **146**, 364-368 (2019)

[27] Ji, C., Levitas, V. I., Zhu, H., Chaudhuri, J., Marathe, A. & Ma, Y. Shear-Induced Phase Transition of Nanocrystalline Hexagonal Boron Nitride to Wurtzitic Structure at Room Temperature and Low Pressure. *Proceedings of the National Academy of Sciences of the United States of America,* **109**, 19108-19112 (2012).

[28] Levitas, V. I., Ma, Y., Hashemi, J., Holtz, M. & Guven, N. Strain-induced disorder, phase transformations and transformation induced plasticity in hexagonal boron nitride under compression and shear in a rotational diamond anvil cell: in-situ X-ray diffraction study and modeling. *Journal of Chemical Physics,* **25**, 044507(1-14) (2006).

[29] Riggs, E & Green, II H. W. A new class of microstructures which lead to transformation-induced faulting in magnesium germanate. *J. Geophys. Res.* **110**, B03202 (2005).

[30] Levitas, V. I. & Shvedov, L. K. Low Pressure Phase Transformation from Rhombohedral to Cubic BN: experiment and theory. *Phys. Rev. B*, **65**, 104109 (2002).

[31] Pandey, K. K. & Levitas, V. I. In situ quantitative study of plastic strain-induced phase transformations under high pressure: Example for ultra-pure Zr. *Acta Materialia,* **196**, 338-346 (2020).

[32] Edalati, K. & Horita, Z. A review on high-pressure torsion (HPT) from 1935 to 1988, *Mat. Sci. Eng. A.,* **652**, 325–352 (2016).

[33] Levitas, V. I., Ma, Y., Selvi, E., Wu, J. & Patten, J. A. High-density amorphous phase of silicon carbide obtained under large plastic shear and high pressure. *Physical Review B*, **85**, 054114 (2012).

[34] Billen, M. I. Deep slab seismicity limited by rate of deformation in the transition zone. *Science Advances* **6**, eaaz7692 (2020).

[35] Cordier, P., Demouchy, S., Beausir, B., Taupin, V., Barou, F. & Fressengeas, C. Disclinations provide the missing mechanism for deforming olivine-rich rocks in the mantle. *Nature,* **507**, 51-56 (2014).

[36] Samae, V., Cordier, P., Demouchy, S., Bollinger, C., Gasc, J., Koizumi, S., Mussi, A., Schryvers, D., & Idrissi H. Stress-induced amorphization triggers deformation in the lithospheric mantle. *Nature,* **591**, 82–86 (2021).

[37] Hirth, G. & Kohlstedt, D. L. Experimental constraints on the dynamics of the partially molten upper-mantle. 2. Deformation in the dislocation creep regime. *J. Geophys. Res.* **100**, 15441–15449 (1995).

[38] Raterron, P., Detrez F, Castelnau, O., Bollinger, C., Cordier, P., & Merkel, S. Multiscale modeling of upper mantle plasticity: From single-crystal rheology to multiphase aggregate deformation. *Physics of the Earth and Planetary Interiors* **228**, 232-243 (2014).

[39] Ogawa M. Shear Instability in a Viscoelastic Material as the Cause of Deep Focus Earthquakes. *J. Geophysical Research,* **92**, 801-810 (1987).

[40] Mohiuddin, A., Karato, S.-I., & Girard, J. Slab weakening during the olivine to ringwoodite transition in the mantle. *Nature Geoscience*, **13**, 170-174 (2020).

[41] Kanamori, H., Anderson, D.L. & Heaton, T.H. Frictional melting during the rupture of the 1994 Bolivian earthquake. *Science* **279**, 839–842, (1998).

[42] Sung, C-M. & Burns R. G. Kinetics of high-pressure phase transformations: implications to the evolution of the olivine $\rightarrow$ spinel transition in the downgoing lithosphere and its consequences on the dynamics of the mantle. *Tectonophysics*, **31**, 1-32 (1976).

[43] Mohiuddin, A. & Karato, S. An experimental study of grain-scale microstructure evolution during the olivine–wadsleyite phase transition under nominally "dry" conditions. *Earth Planet. Sci. Lett.* **501**, 128–137 (2018).

[44] Poirier, J.-P. Introduction to the Physics of the Earth's Interior. *Cambridge University Press* (2000).

[45] Smyth, J. R., Miyajima, N., Huss, G. R., Hellebrand, E., Rubie, D. C., & Frost, D. J. Olivine–wadsleyite–pyroxene topotaxy: Evidence for coherent nucleation and diffusion-controlled growth at the 410-km discontinuity. *Physics of the Earth and Planetary Interiors*, **200-201**, 85–91 (2012).

[46] Zarkevich, N. A., Chen, H., Levitas, V. I. & Johnson D. D. Lattice instability during solid-solid structural transformations under general applied stress tensor: example of Si I $\rightarrow$ Si II with metallization. *Physical Review Letters*, **121**, 165701 (2018).

[47] Levitas, V. I. Phase Transitions in Elastoplastic Materials: Continuum Thermomechanical Theory and Examples of Control. Part I and II. *J. Mech. Phys. Solids,* **45**, 923-947 and 1203-1222 (1997).

[48] Levitas, V. I. Thermomechanical Theory of Martensitic Phase Transformations in Inelastic Materials. *Int. J. Solids and Structures* **35**, 889-940 (1998).

[49] Levitas, V. I., Nesterenko V. F. and Meyers M. A. (1998). Strain-Induced Structural Changes and Chemical Reactions. Part I and II. *Acta Materialia* **46**, 5929-5945 and 5947-5963 (1998).

[50] Levitas, V. I. Structural Changes without Stable Intermediate State in Inelastic Material. Part I and II. *Int. J. Plasticity*, **16**, 805-849 and 851-892 (2000).

[51] Navrotsky, A. Thermodynamic relations among olivine, spinel, and phenacite structures in silicates and germanates: I. Volume relations and the systems NiO-MgO-$GeO_2$ and CoO-MgO-$GeO_2$. *Journal of Solid State Chemistry*, **6**, 21-41, (1973).

[52] Fei, Y. and Bertka, C.M. Phase transitions in the Earth's mantle and mantle mineralogy. In Mantle Petrology: Field Observations and High-Pressure Experimentation. Oxford University Press: Oxford, UK, **6**, 189–207 (1999).

[53] Levitas, V. I., Esfahani, S. E. & Ghamarian I. Scale-free modeling of coupled evolution of discrete dislocation bands and multivariant martensitic microstructure. *Phys. Rev. Letters*, **121**, 205701 (2018).

[54] Gleason, G. & Green, II HW. A general test of the hypothesis that transformation-induced faulting cannot occur in the lower mantle. *Phys. Earth Planet Inter*, **172**, 91–103 (2009).

[55] Takacs, L., Self-sustaining reactions induced by ball milling, *Progress in Materials Science*, **47**, 355-414 (2002).

[56] Zharov, A. A. Reaction of Solid Monomers and Polymers under Shear Deformation and High Pressure. *High Pressure Chemistry and Physics of Polymers*, ed A. L. Kovarskii, Ch. 7, **267-301** (Florida: CRC Press, Boca Raton) (1994)

[57] Koch, C. C., The Synthesis and Structure of Nanocrystalline Materials Produced by Mechanical Attricion: A Review, *Nanostructured Materials*, **2,** 109-129 (1993).

[58] Takacs, L. The historical development of mechanochemistry, *Chem. Soc. Rev.* **42**, 7649-7659 (2013).

[59] Balaz, P, et al. Hallmarks of mechanochemistry: from nanoparticles to technology, *Chem. Soc. Rev.* **42**, 7571-7637 (2013).

[60] Frohlich, C. Deep Earthquakes. Cambridge University Press, Cambridge, UK (2006).

[61] Karato, S., Riedel, M. R. & Yuen, D.A. Rheological structure and deformation of subducted slabs in the mantle transition zone: implications for mantle circulation and deep earthquakes. *Physics of the Earth and Planetary Interiors*, **127**, 83–108 (2001).

[62] Kulnitskiy, B. A., Blank, V. D., Levitas, V. I., Perezhogin, I. A., Popov M., Y., Kirichenko, A. N. & Tyukalova, E. V., Transformation-deformation bands in $C_{60}$ after the treatment in a shear diamond anvil cell. *Materials. Res. Express*, **3**, 045601 (2016).

[63] Markenscoff, X. "Volume collapse" instabilities in deep-focus earthquakes: A shear source nucleated and driven by pressure. *J. Mech. Phys. Solids*, **152**, 104379 (2021).

[64] Levitas, V.I. Idesmanm A.V., Olson, G.B. & Stein, E. Numerical Modeling of Martensite Growth in Elastoplastic Material. *Philosophical Magazine*, A, **82** (3), 429-462 (2002).

[65] Levitas V.I. and Javanbakht M. Phase transformations in nanograin materials under high pressure and plastic shear: nanoscale mechanisms. Nanoscale, **6**, 162 - 166 (2014).

[66] Feng B. and Levitas V. I. Effects of gasket on coupled plastic flow and strain-induced phase transformations under high pressure and large torsion in a rotational diamond anvil cell. *J. Appl. Phys.*, **119**, 015902 (2016).

[67] Levitas, V. I., Ozsoy, I. B. Micromechanical modeling of stress-induced phase transformations. Part 1. Thermodynamics and kinetics of coupled interface propagation and reorientation. *Int. J. Plasticity*, **25**, 239-280 (2009).

[68] Truesdell, C. & Toupin, R.A. The classical field theories. In Handbuch der Physik III/1, ed S. Flügge (Berlin: Springer) (1960).

[69] Ottonello, G., Civalleri, B., Ganguly, J., Zuccolini, M.V., Noel,Y. Thermophysical properties of the $\alpha$–$\beta$–$\gamma$ polymorphs of $Mg_2SiO_4$: a computational study. *Phys Chem Minerals*, **36**, 87–106 (2007).

[70] Liu, M. and Yund, R. A. The elastic strain energy associated with the olivine-spinel transformation and its implications. *Physics of the Earth and Planetary Interiors*, **89**, 177-197 (1995).
